# Efficacy of water application of a humic substance, butyric acid, vitamins C, D, and E and/or electrolytes on performance and mortality in health-challenged nursery pigs¹

**DOI:** 10.1093/tas/txad115

**Published:** 2023-10-02

**Authors:** Michael S Edmonds, Thomas E Weber

**Affiliations:** Nutrition and Product Development, Kent Nutrition Group, Inc., Muscatine, IA 52761, USA; Innovative Solutions, Kent Nutrition Group, Inc., Muscatine, IA 52761, USA

**Keywords:** butyric acid, humic substance, mortality, pig, vitamin C

## Abstract

Health challenges continue to be rampant in nursery pigs which has led to increased industry-wide mortality trends. Therefore, the objective of these three studies was to evaluate a water supplement (**HV**; HydraVantage, Kent Nutrition Group, Muscatine, IA) which is a proprietary blend of a humic substance, butyric acid, and vitamins C, D, and E, as well as an electrolyte blend on nursery pig performance and mortality. Experiment 1 consisted of 196 crossbred weanling pigs (7 pigs per pen with 14 pens per treatment) which were randomly allotted by BW to two treatments consisting of control (water for 33 d) or HV at 15 g/L of stock solution and proportioned through a medicator (1:128) for 11 d followed by water for 22 d. There were no performance differences. However, mortality was reduced (*P* < 0.01) from 6.12% for the control to 0.00% for HV. In experiment 2, there were 488 weanling pigs (6 to 10 pigs/pen with 14 pens per treatment) which were randomly allotted by BW to four treatments in a 34-d trial. Treatment 1 was control (water), and treatments 2 and 4 were HV at 15 g/L of stock solution for 11 and 34 d, respectively. Treatment 3 utilized HV at 15 g/L stock solution during days 0 to 11 with 7.5 g HV/L stock solution utilized during days 11 to 21 followed by water. No performance differences were observed among the four treatments. Mortality was 10.89%, 4.82%, 5.54%, and 7.26% for treatments 1 to 4, respectively, with treatment 1 having a higher mortality (*P* < 0.05) compared to treatments 2 to 4. In experiment 3, a 2 × 2 factorial study was conducted (7 pigs per pen with 14 pens per treatment) in which the treatments were: 1) water; 2) HV at 15 g/L stock solution for 34 d; 3) electrolytes at 241 g/L stock solution for 34 d; and 4) HV at 15 g/L of stock solution and electrolytes at 226 g/L of stock for 34 d. Overall pen gain tended to be improved (*P* = 0.09) with supplemental HV. Moreover, mortality was reduced (*P* = 0.06) by 36% (16.86% mortality for treatments 1 and 3 vs. 10.73% mortality for treatments 2 and 4). Supplemental electrolytes had no effect on mortality. These data suggest that HV has a positive effect by reducing mortality in nursery pigs undergoing health challenges.

## Teaser

Supplementing weanling pigs with HydraVantage, a unique water supplement developed through extensive research, was shown to reduce mortality in health-challenged nursery pigs resulting in potentially a greater proportion of pigs reaching market weight.

## Introduction

Average nursery pig mortality continues to increase (2.97% in 2017 vs. 3.79% in 2021) each year (MetaFarms, 2022). These increases in mortality may be due to numerous ongoing health challenges. A review of infectious diseases (Gebhardt et al., 2020) related to post-weaning mortality, indicates that respiratory and systemic systems were among those most affected. Humic substances have been shown to transfer micronutrients from the soil to plants, increase seed germination rates, and improve the microbial profile in soils (Peña-Méndez et al., 2005), and more recently, the effects of supplementing humic substances in animal and poultry feeds are being reported. In a series of 11 swine growing-finishing studies (Weber and Edmonds, 2022), it was observed that feeding a humic substance, on average, improves livability by about 0.7 units (control with 4.03% mortality vs. added humic substance with 3.37% mortality). In broilers fed diets with an added (0.2%) humic substance for 14 d, and then challenged with *Eimeria* followed by challenge with *Clostridium perfringens*, mortality was significantly reduced by 45% (Edmonds, 2020).

With severe heat stress in broilers 42 d old, feeding a humic substance for 42 d prior to the stressor, decreased mortality by 36% while the combination of the humic substance and a protected butyric acid significantly reduced mortality by 57% (Edmonds et al., 2014). Research in young pigs challenged with *E. coli* lipopolysaccharide (LPS), showed that the combination of a humic substance and a protected butyric acid resulted in a 62% decrease in serum cortisol while IGF-1 was significantly increased by 59% compared to when neither additive was added to the diet (Weber et al., 2014). The use of butyric acid has been shown to reduce liver steatosis and decrease inflammation in animals (Raso et al., 2013) and directly suppress inflammatory responses in numerous cell types (Weber and Kerr, 2006; Ohira et al., 2013).

Supplemental vitamins also help with stressors as shown by added vitamin C significantly improving performance and increasing vitamin D metabolites in health-challenged nursery pigs (Bergstrom and Edmonds, 2014) while added vitamins C and E reduce oxidative stress in heat-stressed poultry (Lin et al., 2006).

While the above studies have involved the supplementation of feeds with these natural additives in challenged pigs and poultry, we are not aware of studies involving the administration of these additives combined in the drinking water of health-challenged nursery pigs. Therefore, our main objective in these experiments was to evaluate water-soluble forms of both a humic substance and butyric acid combined with vitamins C, D, and E to determine the effects on performance and mortality in post-weaning pigs undergoing health challenges. Because electrolytes are often administered via drinking water in stressed pigs post-weaning, we also ­included their supplementation as a second objective in one experiment with health-challenged pigs to determine the effects on performance and mortality.

## Materials and Methods

All research protocols followed the guidelines stated in the Guide for the Care and Use of Agricultural Animals in Agricultural Research and Teaching (FASS, 2010).

### General Procedures

Three experiments were conducted with weanling pigs (initially 20 d of age) that were randomly allotted and blocked by body weight. The pigs were allowed ad libitum access to feed (one self-feeder with four feeder holes) and water (1 cup waterer) in each pen. Pen dimensions were 2.62 m (length) × 1.11 m (width) and, after adjusting for the space occupied by the feeder (0.14 m^2^) and with an average of 7 pigs in each pen (experiments 1 and 3), the floor space per pig was 0.39 m^2^. Experiment 2 had 10 pigs per pen (7 replications), 8 pigs per pen (5 replications), and 6 pigs per pen (2 replications). The pigs were fed commercial diets (Kent Nutrition Group, Muscatine, IA) which were fed in three phases. The diets were in meal form and met or exceeded the nutritional requirements for young pigs (NRC, 2012). Phase 1 diets (days 0 to 11) did not contain butyric acid, a humic substance nor vitamin C. During phase 2 (days 11 to 21), the diets contained a humic substance and a coated butyric acid. In phase 3 (days 21 to 34), the diets contained a humic substance. The proprietary water supplement (HV; HydraVantage®, Kent Nutrition Group, Muscatine, IA) contained a proprietary blend of a freshwater humic substance and butyric acid along with added vitamin C (220,400 mg/kg), vitamin D (2,148,900 IU/kg) and vitamin E (12,893 IU/kg). The electrolytes utilized in experiment 3 consisted of a proprietary mixture of electrolytes, acidifiers, and sugars. The pigs supplied to our research farm had tested positive for porcine reproductive and respiratory syndrome virus (PRRS) and *Streptococcus suis*, which resulted in a disease model to evaluate the proprietary water supplement.

### Animals

Commercial crossbred pigs from the Kent Nutrition Group Research Farm resulting from the cross of DNA males and Fast Genetics females were used in all three experiments.

### Protocol and Design for Experiment 1

A total of 196 pigs were assigned to 1 of 2 treatment groups. There were 14 replicates (pens) per treatment. Treatment 1 was the control (water for 33 d), while treatment 2 utilized HV at 15 g/L of stock solution and proportioned through a Dosatron medicator (1:128) for 11 d followed by water for 22 d. The medications used in phases 1, 2, and 3 were Denagard (35 g/t), Chlortetracycline (400 g/t), and Mecadox (50 g/t), respectively. Phase 1 diets contained 3,150 ppm of added zinc with 3,000 ppm zinc from zinc oxide. Phase 1 to 3 diets contained 205 ppm of added copper from copper sulfate.

### Protocol and Design for Experiment 2

A total of 488 pigs were assigned to 1 of 4 treatment groups. There were 14 replications (pens) per treatment. Treatment 1 was the control (water). Treatments 2 and 4 were HV at 15 g/L of stock and proportioned through a Dosatron medicator (1:128) for 11 and 34 d, respectively. Treatment 3 utilized HV at 15 g/L of stock solution which was proportioned through a medicator (1:128) during days 0 to 11 with 7.5 g/L of stock of HV utilized during days 11 to 21 followed by water. The pigs were diagnosed (serum) with PRRS (RFLP 1-7-4; wild type) by the Iowa State University Diagnostic Laboratory 49 days before this experiment was initiated. The medications used in phases 1, 2, and 3 were Denagard (35 g/t), Chlortetracycline (400 g/t) and Mecadox (50 g/t), respectively. Phase 1 diets contained 3,150 ppm of added zinc with 3,000 ppm zinc from zinc oxide. Phase 1 to 3 diets contained 205, 210, and 210 ppm of added copper via copper sulfate, respectively.

### Protocol and Design for Experiment 3

A total of 396 pigs were assigned to 1 of 4 treatments in a 2 × 2 factorial. There were 14 replications (pens) per treatment. The treatments were: 1) water; 2) HV at 15 g/L of stock and proportioned through a Dosatron medicator (1:128) for 34 d; 3) electrolytes, acidifiers, and sugars utilized at 241 g/L of stock and proportioned through a medicator (1:128) for 34 d; 4) HV at 15 g and electrolytes, acidifiers, and sugars utilized at 226 g/L of stock and proportioned through a medicator (1:128) for 34 d. The medication used in phases 1 to 3 was Denagard (35 g/t). Phase 1 diets contained 3,150 ppm of added zinc with 3,000 ppm zinc from zinc oxide. Phase 1 to 3 diets contained 17, 210, and 210 ppm of added copper via copper sulfate, respectively.

### Statistical Analysis

Pen of pigs served as the experimental unit for all statistical analyses. All three experiments were analyzed as randomized complete block designs using ANOVA. Tukey’s method for all-pairwise comparisons from Statistix 8 (Analytical Software, 2003; Tallahassee, FL) was used in experiments 1 and 2 whereas experiment 3 was analyzed as a 2 × 2 factorial. Differences were considered significant at *P* ≤ 0.05 and a tendency at *P* ≤ 0.10.

## Results

### Experiment 1

While there were no significant differences in performance, pigs in the control treatment group had increased (*P* < 0.06) mortality (3.06%) during days 0 to 11 compared to those pigs with added HV which had no mortality (Table 1). Furthermore, pigs on the control had a mortality of 1.02% and 2.04% during days 11 to 21 and 21 to 33, respectively, while the pigs previously on HV during days 0 to 11 had no mortality. During days 0 to 33, pigs in the control groups had an increase (*P* < 0.05) in mortality (6.12%) when compared to those previously on HV which had no mortality.

**Table 1.** Effect of HydraVantage (HV) on nursery pig performance and mortality, experiment 1^1^

Item	Treatments		
	1	2	Pooled Standard Error Mean (PSEM)
HV (15 g/L stock, days 0 to 11)		√	
Initial body weight, kg	5.70	5.74	
Days 0 to 11			
ADG, g	104	102	6
ADFI, g	153	143	6
G:F	0.68	0.71	0.02
Mortality, %	3.06^c^	0.00^d^	0.81
Days 11 to 21			
ADG, g	390	388	11
ADFI, g	492	484	10
G:F	0.79	0.80	0.02
Mortality, %	1.02	0.00	0.73
Days 21 to 33			
ADG, g	561	569	10
ADFI, g	794	781	14
G:F	0.71	0.73	0.01
Mortality, %	2.04	0.00	0.69
Days 0 to 33			
Pen gain, kg	170.17	182.50	4.08
ADG, g	357	359	4
ADFI, g	489	479	9
G:F	0.73	0.75	0.01
Mortality, %	6.12^a^	0.00^b^	1.14

^1^There were 7 pigs per pen and 14 replications per treatment.

^a,b^Means within rows with different superscripts are different (*P* < 0.05).

^c,d^Means within rows with different superscripts are different (*P* < 0.06).

ADFI, average daily feed intake; ADG, average daily gain; G:F, gain to feed ratio.

### Experiment 2

During days 0 to 11, we did not observe any significant differences in mortality as treatments 2 to 4 had mortality levels below and above the level of the control. While not significant, the overall pig gain was 8.62, 6.64, and 8.43 kg greater for those pigs on HV during days 0 to 11, 0 to 21, and 0 to 34, respectively, compared to those on the control (Table 2). During days 21 to 34, pigs on HV for the entire 34 d had a significantly better G:F (*P* ≤ 0.05) than those in the control group. The average mortality during days 11 to 21 for treatment 2 (HV during days 0 to 11), treatment 3 (HV during days 0 to 11 and 11 to 21), and treatment 4 (HV during days 0 to 34) was 1.07% which was 71.5% lower (*P* < 0.05) than the control at 3.75%. In addition, overall (days 0 to 34) mortality was reduced by 46.1% (*P* < 0.05) when comparing treatments 2 to 4 vs. the control.

**Table 2.** Effect of HydraVantage (HV) on nursery pig performance and mortality, experiment 2^1^

Item	Treatments				PSEM
	1	2	3	4	
HV (15 g/L stock, days 0 to 11)		√	√	√	
HV (7.5 g/L stock, days 11 to 21)			√		
HV (15 g/L stock, days 11 to 34)				√	
Initial body weight, kg	6.67	6.68	6.68	6.68	
Days 0 to 11					
ADG, g	168	171	176	181	13
ADFI, g	232	231	233	240	9
G:F	0.72	0.72	0.74	0.69	0.04
Mortality, %	4.46	1.61	3.93	5.66	1.66
Days 11 to 21					
ADG, g	364	366	374	380	15
ADFI, g	478	484	488	492	17
G:F	0.77	0.76	0.76	0.79	0.02
Mortality^2^, %	3.75	1.61	0.89	0.71	1.17
Days 21 to 34					
ADG, g	505	538	534	544	13
ADFI, g	756	797	784	785	20
G:F	0.67^b^	0.68^ab^	0.69^ab^	0.70^a^	0.01
Mortality, %	2.50	1.61	0.71	0.71	0.85
Days 0 to 34					
Pen gain, kg	89.75	98.37	96.39	98.18	3.18
ADG, g	355	369	371	376	11
ADFI, g	505	522	518	523	14
G:F	0.71	0.71	0.72	0.72	0.01
Mortality^2^, %	10.89	4.82	5.54	7.26	1.98

^1^There were 6 to 10 pigs per pen and 14 replications per treatment.

^2^Treatments 2 to 4 vs. treatment 1 (*P* ≤ 0.05).

^a,b^Means within rows with different superscripts are different (*P* < 0.05).

ADFI, average daily feed intake; ADG, average daily gain; G:F, gain to feed ratio.

### Experiment 3

Mortalities were higher with treatment 2 (HV) and treatment 3 (electrolytes) when compared to the control during days 0 to 11. In addition, during days 11 to 20, we observed numerically lower mortalities for treatments 2, 3, and 4 (HV + electrolytes). Added electrolytes resulted in a significant reduction in G:F during days 11 to 20 (Table 3). Overall (days 0 to 34) pen gain tended to be improved (*P* = 0.09) by 5.23 kg with supplemental HV when compared to those two treatment groups without HV. During days 20 to 34 and overall, treatment 3 had the highest mortality. Supplemental HV resulted in a 47.3% decrease (*P* < 0.06) in mortality during days 20 to 34 with a 36% decrease (*P* < 0.06) in mortality (16.86% mortality for treatments 1 and 3 vs. 10.73% mortality for treatments 2 and 4) during days 0 to 34. In this experiment, mortalities were higher in the later phases compared to experiments 1 and 2 in which mortalities were higher during the first phase.

**Table 3.** Effect of HydraVantage (HV) with and without electrolytes on pig nursery performance and mortality, experiment 3^1^

Item	Treatments				
	1	2	3	4	PSEM
HV (15 g/L stock, days 0 to 34)		√		√	
Electrolytes (241 g/L stock, days 0 to 34)			√		
Electrolytes (226 g/L stock, days 0 to 34)				√	
Initial body weight, kg	5.98	5.89	6.08	5.99	
Days 0 to 11					
ADG, g	125	116	136	128	8
ADFI, g	198	188	213	197	9
G:F	0.62	0.62	0.64	0.66	0.03
Mortality, %	1.02	3.06	3.06	1.02	1.81
Days 11 to 20					
ADG, g	341	332	306	335	16
ADFI, g	412	407	423	427	18
G:F^2^	0.83	0.81	0.70	0.76	0.03
Mortality, %	5.11	3.06	3.06	3.06	1.65
Days 20 to 34					
ADG, g	371	368	350	362	18
ADFI, g	592	558	540	569	22
G:F	0.63	0.63	0.62	0.63	0.02
Mortality^3^, %	9.19	6.13	12.26	5.11	2.62
Days 0 to 34					
Pen Gain^4^, kg	53.84	54.98	47.67	56.99	2.97
ADG, g	284	271	259	275	12
ADFI, g	417	399	404	411	15
G:F	0.68	0.68	0.64	0.67	0.02
Mortality^4^, %	15.32	12.26	18.39	9.19	3.14

^1^There were 7 pigs per pen and 14 replication pens per treatment.

^2^Electrolyte effect (*P* < 0.05).

^3^HydraVantage effect (*P* < 0.06).

^4^HydraVantage effect (*P* < 0.09).

ADFI, average daily feed intake; ADG, average daily gain; G:F, gain to feed ratio.

## Discussion

The swine industry continues to face a multitude of health issues resulting in increased mortality levels that decrease producer profitability. Discovering additives that can work synergistically to help combat stressors in pigs was the key objective of the research shown in this manuscript. We focused on a combination of natural ingredients to use in drinking water as a means of providing support to the pig that may not always consume feed as readily as drinking water when stressed. In summary, we found that a mixture of a proprietary source of freshwater humic substance along with butyric acid and vitamins C, D, and E were helpful in reducing mortality in postweaning pigs that were health-challenged. We have veterinary diagnostic reports that indicate that the sow farm that supplied pigs to our research farm was positive for both PRRS and *S. suis*, pathogens that are associated with increased mortality in pigs postweaning (Gebhardt et al., 2020). While it is common to have greater mortality in the first week postweaning when pigs are undergoing weaning stress and placed in a nursery, this was not always the case in our experiments. Granted in experiments 1 and 2 this did occur, but in experiment 3 we had much greater death losses during the latter two phases, perhaps a result of the severity of disease occurring at a different time point. Another positive aspect of HV involved greater pen weights due to the significant reductions in mortality resulting in a greater amount of weight produced per pen. While we did not see significant responses in body weight gains with HV, we did observe more viable pigs that would potentially reach market weight, thus having positive economic implications for the producer.

There are certainly limitations in the present studies. While we are aware that the pigs were exposed to various pathogens that resulted in substantial mortality, we do not have veterinary reports for each mortality. Having this information would have added to the studies had we requested a veterinary workup for the pigs in each of the three experiments and during the various phases. Furthermore, pathogen challenge studies would be valuable for understanding how the various ingredients performed individually and/or in combination from a mechanistic viewpoint. These areas could involve evaluating parameters such as the microbiome, gut function, and markers of immunity via blood and tissue analysis. Thus, very extensive experiments involving time, laboratory facilities, and funding, with a disease challenge model such as PRRS, would be valuable to better understand mechanisms of action and benefits under specific pathogen challenge scenarios.

Our research with 11 growing–finishing pig trials has shown a reduction in death loss from the added humic ­substance in feed (Weber and Edmonds, 2022). In addition, we observed that this humic substance source was beneficial in broilers undergoing heat stress (Edmonds et al., 2014). In two studies conducted on broilers challenged with ­necrotic enteritis, adding a humic substance increased performance and reduced mortality (Edmonds, 2020) while both feed and water forms of butyric acid also improved performance and decreased mortality (Liu et al., 2018). Moreover, we observed in a commercial layer operation (two barns each with 270,000 layers) undergoing heat stress in the summer, that the layers supplemented with the humic substance and a protected butyric acid had increases in egg ­production compared to layers supplemented with electrolytes (Edmonds, 2012). Furthermore, in grow-finish pigs that had health challenges, we observed that mortality was lowered by supplementing a water-soluble product containing the humic substance and vitamins C, D, and E throughout a 16-wk trial (Edmonds, 2019). Compared to the pig and broiler studies shown above, we did conduct one study in sows in which the added humic substance tended (*P* < 0.18) to increase the number of piglets weaned in a healthy sow herd (Edmonds, 2016).

So how do these natural ingredients function to help animals during time of stress? There are several potential mechanisms with one mode of action involving stabilization of glucose metabolism. In pigs subjected to an inflammatory challenge (LPS), it was observed that blood glucose levels were increased or stabilized when a freshwater humic substance was incorporated into nursery diets for several weeks prior to the stressor (Weber et al., 2014) when compared to pigs fed diets without the humic substance and subjected to LPS.

Another mechanism may be via the adsorption of toxins. In a review article by Trckova et al. (2005), it was reported that humic substances can form chelated complexes with heavy metals, such as lead and mercury, and make them insoluble, thus they are excreted so no adverse effects occur in organisms. In addition, humic substances are highly effective in the adsorption of aflatoxin B_1_ as was found in an in vitro poultry digestive model (Maguey-González et al., 2023).

Decreasing the severity and incidence of gastric abnormalities may be another mechanism via which humic substances can improve livability in pigs. In evaluating the published literature, it appears that gastric abnormalities are present at a high level in cull/dead sows. In one study, involving culled sows from four commercial farms, it was found that gastric ulceration was present in 45% of the sows (Cybulski et al., 2021). In another study on the causes of spontaneous deaths in sows, it was reported that the factors contributing the most to these deaths were heart failures, gastric ulcers, and liver torsion (Monteiro et al., 2022). Another case report (Sanz et al., 2007) on sow mortality found that arthritis was the most common factor in deaths followed by gastric ulcers. In work with AGS cells, humic substances helped decrease inflammation in stomach cells infected with *Helicobacter pylori* which is associated with stomach ulcers (Verrillo et al., 2023). Another report using a rat model showed that humic substances had protective effects on gastric ulcers by alleviating inflammation (Şehitoǧlu et al., 2022).

Modulating the gut microbiota with humic substances is associated with an alleviation of dextran sulfate sodium-induced colitis by increasing the abundance of *Lactobacillus* and *Bifidobacterium* in mice (Huang et al., 2023). In broiler chickens, feeding humic substances resulted in the stability of goblet cells in the jejunum villi from abrupt changes in diets, thus helping to strengthen the mucosal cells in the epithelium of the intestine (López-García et al., 2023). Besides humic substances improving gut health, the source of humic substances in the present paper (Menefee Humate) has also been shown to reduce inflammation and arthritis in rats as demonstrated in a patent issued in 2006 (United States Number 7,067,155 B2).

In broilers, humic substances have been shown to decrease blood heterophil counts (Rath et al., 2006), while in rats, oral administration of potassium humate has been shown to decrease carrageenan-induced paw edema (Naudé et al., 2010) and leonardite humate attenuates the magnitude of the delayed-type hypersensitivity response (Van Rensburg et al., 2007). Mechanistically, humic substances directly suppress the activation of nuclear factor-kappa Β (NF-ĸΒ) by *E. coli* (LPS) in human umbilical cord endothelial cells by preventing the degradation of its inhibitor, IĸΒα (Gau et al., 2000). Moreover, humic substances have stimulated cellular immunity by increasing T-helper lymphocytes in young pigs (Bujňák et al., 2023). In an extensive review by Trckova et al. (2005), beneficial results from supplemental humic substances were observed for immunity, digestion, and growth in chickens, turkeys, pigs, dogs, and cats.

Butyric acid is a short-chain fatty acid that is a very important energy source for cells of the gut. Research by Hammer et al. (2008) showed that butyrate elicits potent beneficial effects on a variety of colonic mucosal functions, such as inhibition of inflammation and decreasing oxidative stress. In broilers, growth has been improved when butyrate was fed prior to a coccidiosis challenge (Leeson et al., 2005). In young pigs, the supplementation with butyric acid (Kotunia et al., 2004; Mazzoni et al., 2008) or a coated calcium butyrate (Claus et al., 2007) resulted in improvements in intestinal morphology. Furthermore, work by Aristimunha et al. (2020) in broilers after 35 d on the test, showed a 32% increase in jejunal villus height from feeding both a humic substance and a coated sodium butyrate.

Several aspects of the immune system appear to be regulated by butyric acid. Liver steatosis and inflammation in rats have been reduced from dietary butyrate (Raso et al., 2013) while inflammatory responses in numerous cell types have been suppressed from butyrate (Weber and Kerr, 2006; Ohira et al., 2013). In research with weanling pigs, Weber and Kerr (2008) observed that coated butyric acid helped regulate the response to inflammatory stimuli in pigs challenged with *E. coli* LPS. Furthermore, other research (Fernández-Rubio et al., 2009) in broilers infected with *Salmonella* Enteritidis showed that sodium butyrate, when partially protected with vegetable fats, provided a greater improvement in reducing infection in the crop, cecum, and liver compared with sodium butyrate without a fat coating. Zhang et al. (2011) reported that broilers fed diets with sodium butyrate showed an inhibition of pro-inflammatory cytokines (serum IL-6 and TNF) in LPS-challenged broilers.

Regarding vitamin C and the immune system, it was reported (Barrio et al., 2019) that adding vitamin C to the drinking water of heat-stressed broilers significantly lowered corticosterone concentration in blood serum.

Another biological mechanism as to how humic substances and butyrate may act synergistically would be via increased activation of the aryl hydrocarbon receptor (AhR). Historically, an increase in AhR activation has been a homeostatic mechanism associated with exposure to toxins such as dioxins. Janošek et al. (2007) observed that five out of seven sources of humic substances were shown to induce AhR-mediated activity. The activation of AhR leads to an upregulation of defense and detoxification mechanisms and enzymes as well as having anti-inflammatory effects. Research by Jourova et al. (2022) showed that butyrate upregulates expression of the AhR gene in a dose-dependent manner in human hepatic cells, thus increasing the drug-metabolizing ability of liver enzymes. Further work with butyrate revealed that gut microbiota-derived endogenous tryptophan metabolites increase the recruitment of AhR to the target gene (Modoux et al., 2022). Taken together, butyrate-mediated increased AhR expression coupled with the presence of an AhR ligand, such as humic substances, may work to synergistically upregulate endogenous defense mechanisms.

The proprietary source of the humic substance used in these experiments is derived from the Menefee Geological Formation in New Mexico. In a patent (United States Number 7,067,155 B2) that was granted in 2006, several key findings were revealed regarding the anti-inflammatory effects of this humic substance source in rat studies. One finding was that when rats were subjected to carrageenan-induced edema, a dietary level of 0.1% to 0.5% of the humic substance resulted in significant reductions in paw edema of 27%. In another study, the use of indomethacin (medication used to inhibit the edema caused by carrageenan) and 0.1% humic substance exhibited a 66.8% inhibition of carrageenan-induced edema, which represented a 28% increase in the anti-inflammatory activity compared to indomethacin alone. Further research showed that arthritis scores could be numerically reduced with the added humic substance. Besides the data in the above patent, in other work (personal communication) with this humic substance in both the insoluble and soluble forms, it was shown after 30 d on treatment to increase white blood cell counts, neutrophils, and lymphocytes from 14% to 57% and after 60 d the increase in neutrophils with the soluble form was 123%. So is there a component in this unique source of humic substance that could explain the effects shown in the patent and the blood work? The researchers have discovered that a specific fungus or fungal spores associated with the Menefee humic substance contain high amounts of biologically natural products. Results have shown that the chemical structure of this fungus is similar to mycophenolate, an agent used as an immunosuppressive drug so this could be one of the key breakthroughs as to the mechanistic activity of this source of humic substance.

A final mode of action of how HV could improve animal resiliency in the face of stressors could involve oxidative stress. Marcinčák et al. (2023) observed that an added humic substance improved the oxidative stability of meat during storage, especially meat with higher fat content. Work from Dyomshyna et al. (2023) reported that adding a humic substance to the water of gerbils increased the activity of catalase, an antioxidant enzyme, and decreased malonic dialdehyde, a prooxidant in liver mitochondria. Work by Lu et al. (2012) showed that butyrate supplementation to gestating sows and piglets enhanced postweaning growth, which was suggested to be mediated by increased substrate oxidation. With regard to vitamins, Lin et al. (2006) observed that supplemental vitamins C and E helped reduce oxidative stress in poultry undergoing excess heat stress. In work with health-challenged nursery pigs (Bergstrom and Edmonds, 2014), supplementing pig diets with a stabilized vitamin C source resulted in improved performance and increased levels of vitamin D metabolites. We believe that the combination of ingredients in HV may play a key role in enhancing oxidative status and immune function and thus showed efficacy in reducing mortality in experiment 3, whereas the addition of electrolytes was without effect.

The plethora of research in this discussion reveals a multitude of mechanisms as to how the components of HV can function in the body and where synergies can occur in helping improve the livability of pigs under various stressors including disease challenges. These natural ingredients are safe and easily mixed in stock solutions, to apply via proportioners, in an efficient manner to pigs during the post-weaning phase. In addition, HV can be applied in an efficient manner with other aspects of swine production where stressors are involved such as lactation and during brief disease and/or heat stress in growing–finishing pigs.
